# A case of bowel entrapment after penetrating injury of the pelvis: don't forget the omentumplasty

**DOI:** 10.1186/1757-7241-19-34

**Published:** 2011-06-10

**Authors:** Ewan D Ritchie, Eelco J Veen, Jan Olsman, Koop Bosscha

**Affiliations:** 1 Department of Surgery, UMC Utrecht, Utrecht, The Netherlands; 2 Department of Surgery, Amphia Hospital, Breda, The Netherlands; 3 Department of Surgery, Jeroen Bosch Hospital, Hertogenbosch, The Netherlands

## Abstract

Bowel entrapment within a pelvic injury is rare and difficult to diagnose. Usually, it is diagnosed late because of concomitant abdominal injuries. It may present itself as an acute intestinal obstruction or, more commonly, as a prolonged or intermittent ileus. Therefore, one should be aware of this late complication and primarily take measures for avoiding bowel entrapment. This report describes an unusual case of bowel entrapment within a pelvic fracture after a penetrating injury, and discusses options for preventing such a complication.

## Introduction

Bowel entrapment within a pelvic injury is rare and difficult to diagnose. Usually, it is diagnosed late because of concomitant abdominal injuries. It may present itself as an acute intestinal obstruction or, more commonly, as a prolonged or intermittent ileus. Therefore, one should be aware of this late complication and primarily take measures for avoiding bowel entrapment.

**Figure 1:**
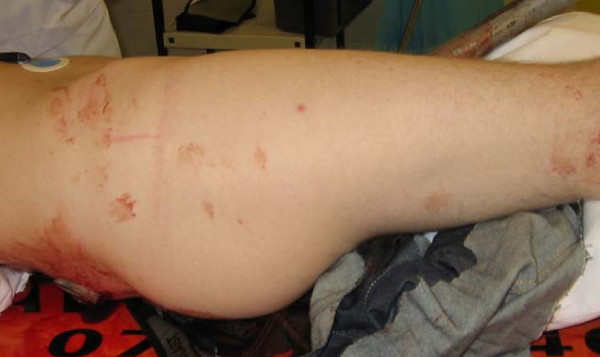
**Patient with metal road work pole presented at the ER at a spine board**.

**Figure 2:**
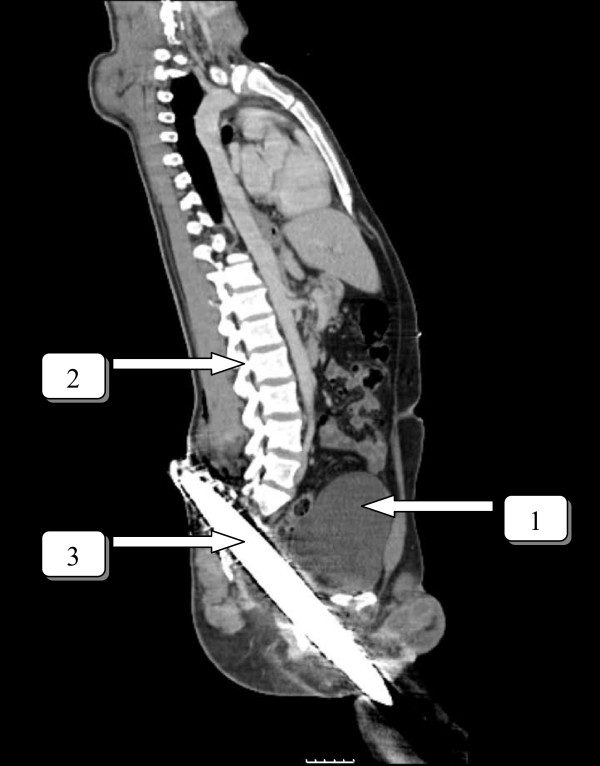
**Coronal view**. Penetrating object caudal to the bladder. 1 bladder. 2 spine. 3 penetrating object.

A twenty-eight year old man was involved in a car crash sustaining a traumatic injury to the lower abdomen. A metal roadwork pole broke and went through the engine and speared the patient. The pole went in at his left groin penetrating his abdomen, and came out on the other side through his sacral bone. (Figure 1) After freeing the patient by cutting the metal pole on both sides, he was transferred to our hospital with the pole in situ. At the emergency department, the patient was examined according to the ATLS principles. The patient had sustained no further damage to the body and was hemodynamically stable. There was no medical or surgical history. There was no neurovascular damage and the function of the perineal region was intact. Trauma radiographs showed the penetrating corpus alienum through the sacral bone. The pelvic ring was intact. A CT scan of the abdomen with contrast confirmed the injury but did not show any bowel or vascular injury (Figure 2).

**Figure 3:**
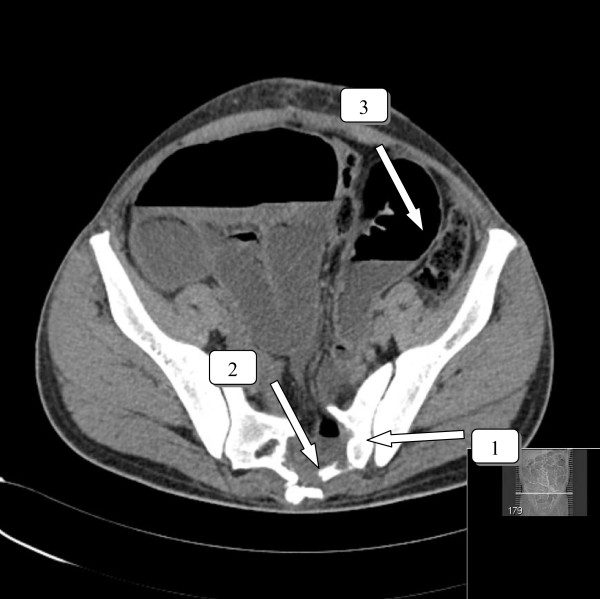
**CT scan with axial view**. Sacral fracture with entrapment and distention of the ileum and jejunum. 1 sacral fracture. 2 distended entrapped ileum 3. distention of the small intestine.

The patient was transferred to the OR and was removed by pulling the pole ventrally without any force. Faecal contamination was diagnosed by exploring the sacral wound. The patient remained hemodynamic stable. An explorative laparotomy was performed and only showed a perforation of the rectosigmoid due to pentrating injury of the pole; a Hartmann's procedure was performed. The central sacral bone defect had a diameter of 2 inches, sparing S1 and S2 foramina and was left untouched. The vascular structures and the urethra were investigated peroperatively, and showed no damage. Postoperatively, the patient went to the ICU. Physical examination postoperatively revealed no new injuries and no neurological deficit. After transfer to the surgical ward, the patient was mobilized. In the early postoperative period, he was diagnosed as having an ileus, which was treated with a nasogastric tube and IV fluids. After 8 days, he showed bowel activity and tolerated fluid intake. Eventually, he was discharged after 21 days. Two weeks after discharge, he presented to the emergency department because of nausea, anorexia and vomiting. He had lost 10 kg in weight. His stomy had been intermittent productive. Physical examination revealed a normal temperature and a regular pulse. The abdomen was nontender with distention and showed a paucity of bowel sounds. Laboratory tests were unremarkable. He was admitted and received a nasogastric tube and IV drip. A CT scan showed a significant distention of the ileum and jejunum, demonstrating an obstruction in the pelvic area (Figure 3). At laparotomy, an obstructed distented small intestine was seen due to a segment of small intestine herniated in the perforated sacral bone. Eventually, an end-ileostomy was installed because of the distention of the small intestine and, also, an omentoplasty was performed to fill up the defect in the sacral bone. The postoperative period was seriously complicated by a pulmonary embolism which was treated with anticoagulants, although the patient received a low molecular weight heparin during the first six weeks after the trauma. The complication was probably caused by prolonged bedrest. A normal diet was started soon. At follow-up, he had gained sufficient weight. Restorative surgery will be planned in the near future.

## Discussion

Penetrating trauma to the abdomen can cause severe injuries to multiple organs, but entrapment of the bowel within a pelvic fracture is rare.

In case of bowel entrapment in a fracture, there must be a substantial displacement of the fracture and disruption of tissue. Bowel entrapments have been recorded occasionally in sacral, iliac wing and acetabular fractures. A paralytic ileus is a known complication of abdominal surgery and a prolonged recovery is common. However, symptoms can mask true mechanical obstruction. A paralytic ileus occurs in 5.5 to 18 percent of pelvic fractures, lasting an average of 2.6 days [1-3]. Literature shows that the diagnosis is delayed by an average of two weeks, presumably due to difficulty in differentiating entrapment from the more common paralytic ileus. Therefore, entrapment of the small bowel can be easily overlooked when the potential cause of symptoms are not recognized. If an ileus with a pelvic fracture persists for a lengthy period of time, an occult bowel injury such as entrapment at the fracture site should be considered. Radiological techniques can be useful in making the diagnosis. Plain radiographs can be helpful in identifying obstructions. Oral contrast studies can be misleading due to normal transit times for the passage of contrast, even in case of a herniated bowel. A CT scan with enteric contrast can demonstrate a herniated or entrapment bowel in the fracture [4,5].

To treat the problem and avoid recurrent obstruction an omentoplasty was performed to seal the pelvic cavity. The use of the greater omentum in the pelvic cavity was first described for repair of fistulas in the genitourinary tract. Since then, different use of omentum have been promoted in healing in a range of applications including closure of peptic ulcers, management of empyemas, infected thoracotomy wounds and wounds following excision of radionecrosis [6,7]. In our case, the fractured sacral bone created a "dead space" as seen also in case of perineal wounds and/or the presence of a presacral dead space after an abdominoperianeal resection. We prefer filling the "dead space" with an omentumplasty, above a bonegraft filling, as we were performing a laparotomy. Although an autologic bonegrafting is optional. The use of the omentum exludes the small intestine from the pelvic area, and should have been performed primarily to prevent the bowel entrapment.

## Conclusion

Bowel entrapment within a pelvic fracture is rare and hard to diagnose. Usually, it is diagnosed late because of concomitant abdominal injuries. To prevent the problem and avoid recurrent obstruction an omentoplasty should be performed to seal the pelvic cavity during the primary procedure.

## Consent

There was informed consent of the patient obtained for publication of this case report and accompanying images.

## Competing interests

The authors declare that they have no competing interests.

## Authors' contributions

ER: Participiating in design of the study, the sequence alignment and draft of the manuscript. EV: Participiating in design of the study, the sequence alignment and draft of the manuscript. JO: Participated in design and coordination of the case. KB: Participated in design and coordination of the case. All authors read and approved the final manuscript

## References

[B1] Buchanan J Bowel entrapment by pelvic fracture fragments: a case report and review of the literature Clin Orthop Related Res 1980 147 164 6 7371287

[B2] Hurt B Oschner L Schiller W Prolonged ileus after severe pelvic fracture Am J Surg 1983 146 755 7 10.1016/0002-9610(83)90334-3 6418021

[B3] Levine J Crampton R Major abdominal injuries associated with pelvic fractures Surg Gynecol Obstet 1963 116 223 6 13930147

[B4] Crowther A McMaster J Abercrombie J Hahn D Sacral fracture associated with small bowel entrapment: A case report J Orthop Trauma 2006 20 580 3 10.1097/01.bot.0000245002.75621.ff 16990732

[B5] Stubbart J Merkley M Bowel entrapment within pelvic fractures:a case report and review of the literature J Orthop Trauma 1999 13 145 8 10.1097/00005131-199902000-00014 10052792

[B6] Nilsson P Omentoplasty in abdominoperineal resection A review of the literature using a systemic approach Dis Colon Rectum 2006 49 1354 61 10.1007/s10350-006-0643-x 16897330

[B7] O'Leary D Use of the greater omentum in colorectal surgery Dis Colon Rectum 1999 42 533 9 10.1007/BF02234183 10215058

